# Kalemia Significantly Influences Clinical Outcomes in Patients with Severe Traumatic Brain Injury (TBI)

**DOI:** 10.3390/diagnostics15151878

**Published:** 2025-07-26

**Authors:** Bharti Sharma, Munirah Hasan, Usha S. Govindarajulu, George Agriantonis, Navin D. Bhatia, Jasmine Dave, Juan Mestre, Shalini Arora, Saad Bhatti, Zahra Shafaee, Suganda Phalakornkul, Kate Twelker, Jennifer Whittington

**Affiliations:** 1 Trauma Unit, Department of Surgery, NYC Health and Hospitals—Elmhurst, Elmhurst, NY 11373, USA; hassanm14@nychhc.org (M.H.); agriantg@nychhc.org (G.A.); bhatian1@nychhc.org (N.D.B.); davej@nychhc.org (J.D.); mestreju@nychhc.org (J.M.); arorash@nychhc.org (S.A.); bhattisa@nychhc.org (S.B.); shafaeez1@nychhc.org (Z.S.); phalakos@nychhc.org (S.P.); twelkerk1@nychhc.org (K.T.); harrisj20@nychhc.org (J.W.); 2 Trauma Unit, Department of Surgery, Icahn School of Medicine at Mount Sinai, New York, NY 10029, USA; 3 Center of Biostatistics, Department of Population Health Science and Policy, Icahn School of Medicine at Mount Sinai, New York, NY 10029, USA; usha.govindarajulu@mountsinai.org

**Keywords:** potassium level, hypokalemia, traumatic brain injury, injury severity score, severe trauma

## Abstract

**Objective**: Potassium levels (KLs) influence clinical outcomes in severe traumatic brain injury (TBI). This study investigates the relationship between KLs and clinical outcomes to improve prognosis and guide management. **Method**: A retrospective study was conducted at a level 1 trauma center in Queens, New York, from January 2020 to December 2023. Patients with an AIS score of 3 or higher were included. KLs were measured at the time of hospital admission, ICU admission, ICU discharge, hospital discharge, and death, if applicable. Clinical outcomes such as age, race, length of hospital stay (H LOS), ICU length of stay (ICU LOS), ventilation days (VDs), Glasgow Coma Scale (GCS), and mortality were assessed. **Results**: KLs were categorized into five groups: extreme hypokalemia (<2.5 mEq/L), hypokalemia (2.6–3.5 mEq/L), normokalemia (3.5–5.2 mEq/L), hyperkalemia (5.2–7.0 mEq/L), and extreme hyperkalemia (>7.0 mEq/L). Significant correlations were observed between KLs at hospital admission and age (*p* = 0.0113), race (*p* = 0.003), and H LOS (*p* = 0.079). ICU KLs showed positive correlations with AIS head score (*p* = 0.038), ISS (*p* = 7.84 × 10^−6^), and GCS (*p* = 2.6 × 10^−6^). ICU KLs were also associated with LOS in the Emergency Department (ED) (*p* = 6.875 × 10^−6^) and ICU (*p* = 1.34 × 10^−21^), as well as VDs (*p* = 7.19 × 10^−7^). ICU discharge KLs correlated with ISS (*p* = 2.316 × 10^−3^), GCS (*p* = 2.201 × 10^−3^), ED LOS (*p* = 3.163 × 10^−4^), and VDs (*p* = 7.44 × 10^−4^). KLs at discharge were linked with mortality (*p* < 0.0001) and H LOS (*p* = 0.0091). Additionally, KLs at the time of death were correlated with ISS (*p* = 0.01965), GCS (*p* = 0.01219), ED LOS (*p* = 0.00594), ICU LOS (*p* = 0.049), VDs (*p* = 0.00005), and mortality (*p* < 0.0001). **Conclusions**: Potassium imbalances, especially hypokalemia, significantly affect outcomes in severe TBI patients. Monitoring and managing KLs may improve prognosis.

## 1. Introduction

Traumatic brain injury (TBI) represents a major global health challenge, being a leading cause of both death and disability resulting from trauma. In the United States, TBI accounts for approximately 52,000 deaths annually [1,2], imposing a significant economic burden with treatment and care costs reaching an estimated USD 48.3 billion per year [1,2]. The consequences of TBI extend beyond mortality, leading to a variety of complications, including seizures, hydrocephalus, vascular injuries, multiple organ dysfunction, significant fluid, deep vein thrombosis, coagulopathies, and electrolyte imbalance [3].

Electrolyte disturbances, particularly involving potassium, have garnered increasing attention in recent years. While most studies have emphasized sodium dysregulation, potassium imbalances—both hypokalemia and hyperkalemia—are increasingly recognized in trauma populations [4,5,6,7]. Potassium is a vital intracellular cation involved in membrane potential maintenance, muscle contraction, and acid–base homeostasis. Disruption in potassium regulation following TBI may result from catecholamine surges, renal losses, or treatment-related factors. In circumstances of TBI, this regulation of potassium can be disrupted, leading to hypokalemia and hyperkalemia. Electrolyte disturbances, including potassium imbalances, are found in up to 50% of trauma cases [8]. Up to 45% of TBI patients develop hypokalemia [7,9,10], which can lead to complications such as arrhythmias, rhabdomyolysis, renal failure, and hyperglycemia [11,12]. Although less common, hyperkalemia is also observed—particularly early in resuscitation—and may reflect hemolysis, acidosis, or pharmacologic triggers [13,14,15,16,17]. Both conditions may co-occur or evolve dynamically during hospitalization [4,15,17,18,19,20].

Standard thresholds for potassium classification vary slightly across the literature [6,10,21,22,23,24]. In our study, we define potassium ranges as follows: extreme hypokalemia (<2.5 mEq/L), hypokalemia (2.6–3.5 mEq/L), normokalemia (3.5–5.2 mEq/L), and hyperkalemia (5.2–7.0 mEq/L).

Despite extensive documentation of dyskalemia incidence, less is known about how potassium levels (KLs) fluctuate during hospitalization and how these fluctuations relate to clinical outcomes. Identifying robust prognostic markers in TBI is essential for improving decision-making, targeting interventions, and optimizing resource use [25].

We hypothesize that fluctuations in KLs, especially severe hypokalemia, are linked to worsened clinical outcomes in TBI patients. The primary objective of this study is to examine the relationship between KLs and key clinical predictors, such as the Injury Severity Score (ISS), the initial Glasgow Coma Scale (GCS) score at the time of emergency room admission, and various outcomes, including mortality, length of hospital stay (LOS), ICU duration, and mechanical ventilation requirements. By investigating potassium as a potential biomarker, this study aims to highlight its critical role in optimizing patient care and improving outcomes for individuals with severe TBI.

In addition to trauma-related physiological disturbances, chronic comorbidities such as diabetes mellitus and hypertension—common in older adults—may also influence potassium homeostasis. However, these variables were not included in our dataset and remain an important area for future investigation.

## 2. Method

### 2.1. Study Population

This is a single-center retrospective review conducted at a level 1 trauma center verified by the American College of Surgeons in Queens, New York City, NY, USA. We included all patients who presented with a severe TBI between 1 January 2020 and 31 December 2023. All patients with an Abbreviated Injury Severity (AIS) score of 3 or higher were included. Patients who were discharged or transferred and had KLs collected were also included in the analysis. We excluded patients who had a COVID-19 infection at the time of their injury, who died or were discharged within 24 h of their initial injury, and has an AIS less than 3 (minor and moderate injuries). We found a total of 1040 patients with severe TBIs, and 981 total patients were included in the study. This study received approval from the institutional review board (IRB) at Elmhurst Facility, with IRB number 24-12-092-05G.

Data for all patients with severe TBI were requested from the National Trauma Registry of the American College of Surgeons (NTRACS) database at our center, Elmhurst Hospital Center. NTRACS gathers various categories of TBIs. To maintain clarity in our table and results, we have included only the combinations of TBIs that are relevant to our study. When necessary, we reviewed the patient’s medical charts to collect all pertinent information required for this research. Patients were identified based on the injury mechanism, cause of injury, primary mechanisms, International Classification of Diseases (ICD9 or ICDL0 E-Code), and AIS (head). The AIS ranges from 1 to 6 per body region. Severe TBI is defined by a GCS of 8 or less after resuscitation, but before sedation is given to the patient. In our trauma center, endotracheal intubation was routinely performed for patients with a GCS ≤ 8 using standard Advanced Trauma Life Support (ATLS) protocols. Intubation was also indicated for patients with compromised airways, persistent hypoxia, or severe facial trauma. Intracranial pressure (ICP) monitoring was placed in patients with severe TBI at the discretion of the consulting neurosurgeon. Operative interventions, such as craniotomy or decompressive craniectomy, were conducted when indicated, based on radiologic findings and neurosurgical evaluation. KLs were categorized into five ranges, extreme hypokalemia (less than 2.5 mEq/L), hypokalemia (2.6–3.5 mEq/L), normokalemia (3.5–5.2 mEq/L), hyperkalemia (5.2–7.0 mEq/L), and extreme hyperkalemia (greater than 7.0 mEg/L), and we studied if there were any statistically significant differences in KL at hospital admission (TA), ICU admission (RL1), ICU discharge (RL2), hospital discharge (HD), and at patient death (PD) across various clinical outcomes. The clinical outcomes studies were ED, ICU, Hospital LOS, mechanical ventilation time, and mortality.

Data regarding potassium supplementation (e.g., intravenous or oral replacement therapy) were not uniformly recorded in the trauma registry or extracted from patient charts. Therefore, we were unable to analyze the impact of potassium correction on patient outcomes in this retrospective cohort.

### 2.2. Data Collection

We collected data using a data collection tool (Excel sheet or spreadsheet). We incorporated all data elements into this tool, for example, patient demographics, clinical outcomes, and biochemical data. Baseline admission data included data elements like demographics (age, sex, race, ethnicity), the AIS, and the ICD injury description. Age ranges were divided into groups: pediatric (less than 15), young adults (ages 15–25), older adults (ages 24–64), and elderly (ages greater than 65). Sex was categorized as male and female. We also included the types of injury: blunt vs. penetrating.

Further subset analysis was performed based on the unique intracranial ICD-10 codes assigned to each patient. The potassium was obtained manually from charts at four pre-determined time frames of a patient’s hospital stay. These were hospital admission, ICU admission, ICU discharge, and then either death or hospital discharge. “Admission” was defined as the first measured level of a metabolite after admission to the trauma bay. “ICU admission” was defined as the first measured level of a metabolite after admission to the ICU. At the same time, “ICU discharge” was the last measured level of a metabolite before arrival in a step-down or floor unit. “Hospital discharge” was the last measured value for a metabolite before discharge, and “death” was the last measured value before the time of death. In a few cases, missing data were filled in by taking the most recent potassium measurement in a given defined time frame [1,2,4,5,6,7,9,10,11,12], even if that potassium value was used for another time frame. For example, if a patient was discharged with only one set of lab values obtained, the KL that was used for admission was also used for discharge, as that was technically the first KL obtained during hospital admission and the last obtained before hospital discharge. Given the universal use of basic metabolic panels and blood gases, the amount of missed potassium values, while not counted, was negligible for our study. The primary outcome variable in this dataset was mortality. Secondary outcomes were ED LOS, hospital LOS, ICU LOS, and days requiring mechanical ventilation. All secondary outcomes were measured in days, and mortality was assigned a binary variable: 0 for no mortality recorded, and 1 if a patient had a recorded mortality. To convert KLs to a categorical variable for these analyses, patients were assigned a potassium range based on accepted levels in the literature [10,21,22]. KLs were categorized into five clinical ranges (see Section 2).

### 2.3. Statistical Analysis

Pearson Chi-square tests were conducted to test the association between certain categorical variables. The Kruskal–Wallis test was used to test if various scores and lengths of stays differed between different K levels for K measured at different points of stay. The analyses were conducted in SAS Version 9.4. A significance level of 0.05 was used for all analyses.

### 2.4. Results

A total of 981 patients were included in the study after applying the exclusion criteria. Among these patients, 752 were male and 229 were female. The age distribution was as follows: 17 patients were in the pediatric group (from 0 to less than 15 years old), 60 in the younger adult group (from greater than or equal to 15 to less than 24 years old), 579 in the older adult group (from greater than or equal to 24 to less than or equal to 64 years old), and 310 in the elderly group (from greater than or equal to 65 years old). The average age of all participants was 53 years. Of the reported injuries, 961 were due to blunt trauma, while 20 were classified as penetrating trauma. Moreover, 126 patients in our study died, and 855 patients survived their index hospitalization. Table 1 of this paper represents the baseline demographics of the patients included in this study.

Our comprehensive analysis categorized KLs into five distinct ranges (see Section 2) based on established clinical thresholds [10,21,22,23]. This categorization provided valuable insights into the relationship between potassium imbalances and various clinical outcomes.

KLs were measured at key stages of the hospital stay: admission (TA), ICU admission (RL1), ICU discharge (RL2), hospital discharge (HD), and patient death (PD). The results reveal a significant shift in KL as patients progress through their hospitalization, particularly among those admitted to the ICU. In our study, we found that the average length of hospitalization was 12 days, the average ICU stay was 4 days, and the average length of ventilator use was 2 days. Table 2 presents the frequency, percentage, cumulative frequency, and cumulative percentage of various KLs at each time point.

Upon hospital admission (TA), most patients (79.71%) had normal KLs, with 12.03% experiencing hypokalemia and 4.28% exhibiting extreme hypokalemia. Only a small percentage (3.98%) showed hyperkalemia. However, by the time patients entered the ICU (RL1), there was a drastic shift in KLs. Extreme hypokalemia rose sharply to 39.14%, while hypokalemia decreased to 7.75%. Normokalemia was still observed in 51.07% of patients, but hyperkalemia was significantly reduced to 2.04% (Table 2).

As patients progressed to ICU discharge (RL2), extreme hypokalemia increased further to 42.71%, while hypokalemia dropped to 3.77%. Normokalemia remained relatively stable at 52.80%, and hyperkalemia decreased even further to 0.71%. At hospital discharge (HD), extreme hypokalemia accounted for 28.03% of patients, with hypokalemia in 2.45% and normokalemia in 68.60%. Hyperkalemia remained low at 0.92% (Table 2).

The data also revealed that, at the time of death (PD), 91.95% of patients had extreme hypokalemia, while only 0.82% had hypokalemia, 4.69% had normokalemia, and 1.43% exhibited extreme hyperkalemia. These findings suggest that as patients progress through their hospitalization, potassium imbalances, particularly extreme hypokalemia, become more prevalent, and extreme hypokalemia is strongly associated with patient mortality (Table 2).

The overall percentages for all time points (hospital admission, ICU admission, ICU discharge, hospital discharge, and patient death) show that extreme hypokalemia occurred in 49.03%, hypokalemia in 38.74%, normokalemia in 11.82%, and hyperkalemia in 0.31%. These results emphasize the prevalence of severe hypokalemia and hypokalemia as the primary pathologies in TBI patients (Table 2).

The mean hospital LOS (LOS) for patients with various KLs (extreme hypokalemia, hypokalemia, normokalemia, hyperkalemia, and extreme hyperkalemia) was compared at different time points during hospitalization, including hospital admission, ICU admission, ICU discharge, hospital discharge, and patient death (Table 3).

At hospital admission, the mean LOS was highest in the hypokalemia group (13.16 days), followed by the hyperkalemia group (12.33 days), the normokalemia group (11.72 days), and the extreme hypokalemia group (5.29 days) (Table 3).

At ICU admission, the mean LOS was highest in the hypokalemia group (14.05 days), followed by the normokalemia group (13.24 days), the hyperkalemia group (12.15 days), and the extreme hypokalemia group (9.06 days) (Table 3).

At ICU discharge, the hyperkalemia group had the longest mean LOS (21.57 days), followed by the normokalemia group (14.21 days) and the extreme hypokalemia group (8.78 days) (Table 3).

At hospital discharge, the normokalemia group had the longest LOS (12.42 days), followed by the hyperkalemia group (11.44 days), the extreme hypokalemia group (10.33 days), and the hypokalemia group (4.92 days) (Table 3).

At the time of patient death (KPD), the hypokalemia group had the longest mean LOS (35.38 days), followed by the hyperkalemia group (19 days), the extreme hypokalemia group (11.51 days), the normokalemia group (10.32 days), and the extreme hyperkalemia group (5.86 days) (Table 3). It is noteworthy that extreme hypokalemia (KL higher than 7) was observed only in patients who died and was not seen at any other time point.

In this study, various prognostic variables (ISS, initial GCS, ED LOS, hospital LOS, ICU LOS, and ventilatory days) were compared at the different times during which KLs were collected during hospitalization. These time points included hospital admission (KTA), ICU admission (KRL1), ICU discharge (KRL2), hospital discharge (KHD), and the time of patient death (KPD). The median ISS ranged from 16 to 18, with a quartile range from 14 to 15 across the various time points when potassium was collected. The median GCS recorded in the ED remained at 15, with a quartile range from 2 to 8 during the different collection times. The median ED LOS (days) was between 8.84 and 9.97, with a quartile range from 9.44 to 14.30 at the different time points of potassium collection. The median hospital LOS (days) ranged from 5 to 7, with a quartile range from 11 to 13 across the times potassium was collected. The median ICU LOS (days) ranged from 1.28 to 2.2, with a quartile range from 3.84 to 4.91 at the various collection points. The median number of ventilatory days was 0 for all the time points at which potassium was collected.

This study found multiple significant relationships between KLs and various prognostic factors and outcomes. Several statistical methods were employed to analyze these relationships, including the Kruskal–Wallis test and the Chi-square test. These tests were used to compare variance across different groups, particularly when the data was not normally distributed. KLs were measured at key points: hospital admission, ICU admission, ICU discharge, hospital discharge, and patient death for those who applied. Clinical outcomes such as age, race, H LOS, ICU LOS, ventilation days (VDs), GCS, and mortality were assessed to examine their correlation with KLs.

At hospital admission (TA), KLs were significantly associated with demographic factors such as patient age and race. Most patients in our analysis were between 24 and 64 years old, and we observed a significant difference in KLs based on age with a Chi-square statistic (χ^2^) of 21.32, sample size of 966, and *p*-value of 0.011. Significant differences in KLs were also found across different racial groups (χ^2^ = 38.64, df = 18, sample size = 981, *p* = 0.003), suggesting that race may influence potassium regulation. No significant variation in KLs was observed between male and female patients at admission. Moreover, a near-significant trend was found between KLs at admission and hospital LOS (H LOS), with a Kruskal–Wallis statistic (H) of 11.87 and a *p*-value of 0.079, suggesting that patients with more extreme potassium imbalances at admission may experience longer hospitalizations.

**Figure 1:**
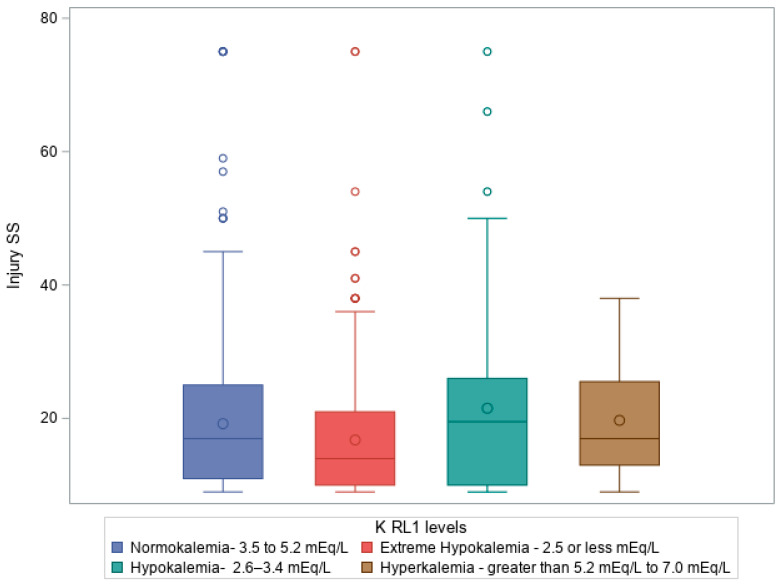
Box plot illustrating Kruskal–Wallis test comparing ISS on admission compared to KLs on admission to ICU (K RL1 levels), *p* = 7.84 × 10^−6^.

When examining KLs at trauma service admission (RL1), typically corresponding to ICU admission, our analysis revealed a strong positive correlation between KLs and various injury severity scores, including the AIS head, with a Chi-square statistic (χ^2^) of 17.75, sample size = 981, and *p*-value of 0.038. The Kruskal–Wallis test also revealed significant differences in KLs at ICU admission regarding ISS and GCS total score, with Kruskal–Wallis statistics (H) of 26.408 (*p* = 7.84 × 10^−6^) and 28.694 (*p* = 2.6 × 10^−6^), respectively. Figure 1 shows a box plot illustrating a Kruskal–Wallis test comparing ISS on admission compared to KL on admission to ICU (RL1 levels), *p* = 7.84 × 10^−6^, where patients with hypokalemia had high ISS, followed by normokalemia, hyperkalemia, and then extreme hypokalemia.

**Figure 2:**
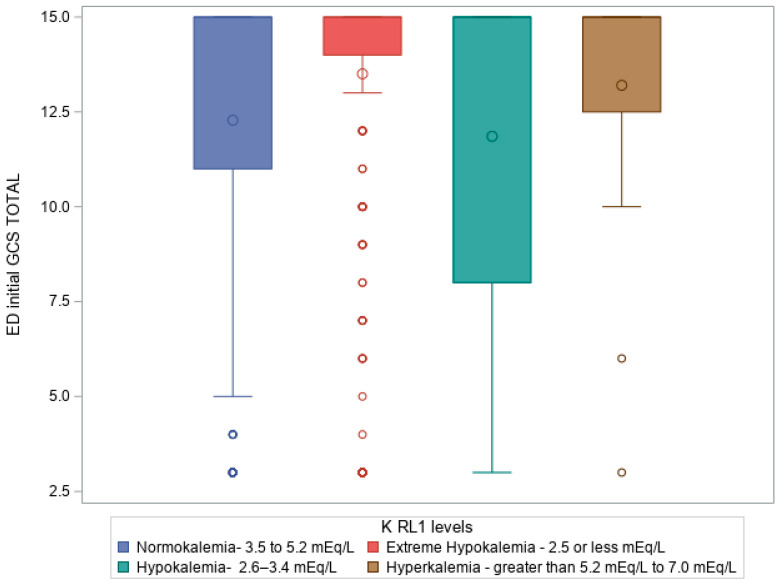
Box plot illustrating Kruskal–Wallis test comparing ED initial GCS total to KLs on admission to ICU (K RL1 levels), *p* = 2.6 × 10^−6^.

Figure 2 shows a box plot illustrating a Kruskal–Wallis test comparing ED initial GCS total to KL on admission to ICU (RL1 levels), *p* ≤.0001, where patients with hypokalemia had a predominant frequency of patients with lower GCS in ED, followed by normokalemia, extreme hypokalemia, and, lastly, hyperkalemia.

Furthermore, KLs in the ICU were significantly associated with extended stays in both the emergency department (ED) (H = 21.80, df = 3, *p* = 6.87 × 10^−6^) and the ICU (H = 100.305, df = 3, *p* = 1.34 × 10^−21^), as well as the number of VDs required (H = 31.35, df = 3, *p* = 7.19 × 10^−7^). These results suggest that potassium imbalances may not only reflect disease severity, but also correlate with prolonged care needs, such as longer stays in critical care units and mechanical ventilation.

**Figure 3:**
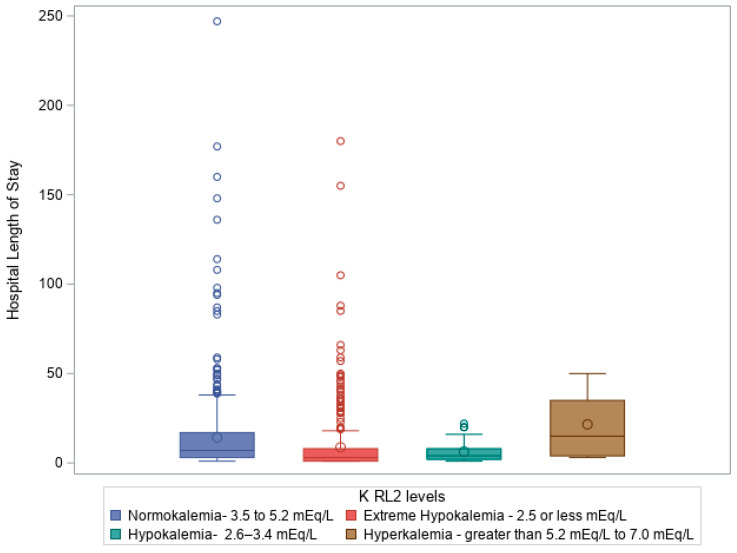
Box plot illustrating Kruskal–Wallis test comparing hospital LOS (days) to KLs on ICU discharge (K RL2 levels), *p* = 1.33 × 10^−15^.

Similarly, a Kruskal–Wallis test revealed a significant difference in KLs at ICU discharge (RL2) regarding ISS (H = 14.48, df = 3, *p* = 0.002) and GCS (H = 14.59, df = 3, *p* = 0.002). KLs at ICU discharge were also significantly linked to extended stays in the ED (H = 18.68, df = 3, *p* = 0.0003), hospital LOS (H = 72.36, df = 3, *p* = 1.33 × 10^−15^), ICU LOS (H = 91.17, df = 3, *p* = 1.23 × 10^−19^), and VDs (H = 16.89, df = 3, *p* = 0.0007). Figure 3 shows a box plot illustrating a Kruskal–Wallis test comparing hospital LOS (days) to KLs on ICU discharge (RL2 levels), *p* ≤ 0.0001, showing a significant impact from KLs on H LOS.

**Figure 4:**
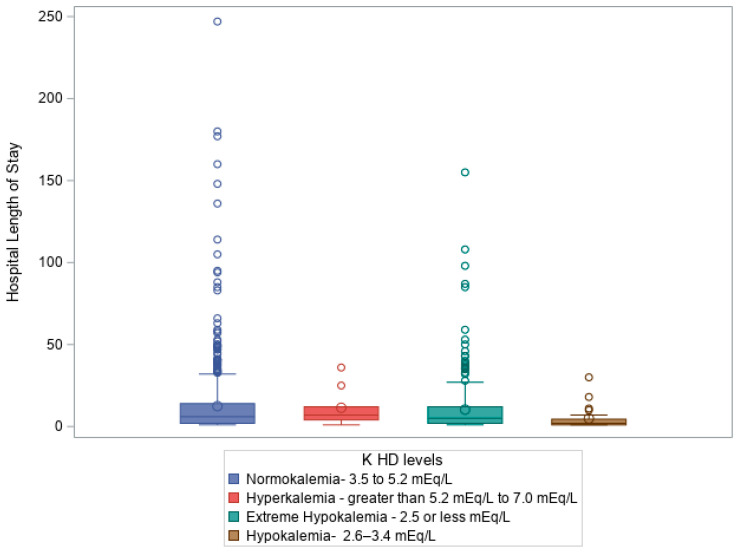
Box plot illustrating Kruskal–Wallis test comparing hospital LOS (days) to KLs on hospital discharge (K HD levels), *p* = 0.009.

At hospital discharge (HD), KLs were strongly correlated with patient mortality, with a Chi-square statistic (χ^2^) of 24.323, three degrees of freedom (df), and a *p*-value < 0.0001, Additionally, KLs at discharge were significantly associated with H LOS (H = 11.56, df = 3, *p* = 0.009), suggesting that patients with longer hospital stays may experience greater disturbances in potassium balance, potentially due to prolonged illness, treatments, or complications. Figure 4 shows a box plot illustrating a Kruskal–Wallis test comparing hospital LOS (days) to KLs on hospital discharge (K HD levels), *p* =0.009.

The analysis also highlighted KLs at the time of patient death, which were significantly correlated with several critical clinical factors, including ISS (H = 11.71, df = 4, *p* = 0.020), GCS (H = 12.82, df = 4, *p* = 0.012), ED LOS (H = 14.47, df = 4, *p* = 0.006), ICU LOS (H = 9.55, df = 4, *p* = 0.049), and VDs (H = 25.00, df = 4, *p* = 5 × 10^−5^). KLs at the time of patient death (PD) were strongly correlated with mortality, with a Chi-square statistic (χ^2^) of 98.32 and a *p*-value < 0.0001. These findings highlight the fact that severely ill patients, especially those requiring extended ICU stays and prolonged mechanical ventilation, are more likely to experience extreme potassium imbalances.

This study demonstrates a significant association between potassium imbalances, particularly extreme hypokalemia, and critical clinical outcomes in trauma patients. As patients progress through their hospitalization, potassium disturbances, especially extreme hypokalemia, become more prevalent and are strongly correlated with patient mortality. The findings further suggest that potassium imbalances are linked to injury severity, GCS, and prolonged ICU and hospital stays. Given these associations, monitoring KLs could serve as a valuable prognostic tool in critically ill trauma patients, guiding treatment decisions and improving patient outcomes.

## 3. Discussion

### 3.1. Key Findings

This single-center study highlights a significant association between potassium imbalances—particularly extreme hypokalemia—and adverse clinical outcomes in patients with severe TBI. Notably, the prevalence of dyskalemia increased throughout hospitalization, with nearly half of all patients experiencing extreme hypokalemia by ICU discharge, with the most severe potassium imbalances observed at the time of death.

Although normokalemia predominated at **hospital admission**, a substantial proportion of patients exhibited hypokalemia or extreme hypokalemia. Table 2 provides an overview of the distribution of KLs at this stage, showing that while most patients had normal potassium, a considerable percentage had abnormal levels, with hypokalemia and extreme hypokalemia being more common. These early imbalances were significantly associated with demographic factors such as age and race, suggesting that certain populations may be more susceptible to potassium dysregulation following TBI.

**By ICU admission**, potassium profiles shifted markedly, with a significant rise in extreme hypokalemia. Figure 1 illustrates the association between higher ISS and KL, with more severe injuries correlating with greater potassium derangements. Similarly, Figure 2 demonstrates that patients with a lower GCS were more likely to present with hypokalemia or extreme hypokalemia. These findings underscore the close link between injury severity and potassium imbalance in the early critical care phase.

By **ICU discharge**, the prevalence of extreme hypokalemia increased further, while hypokalemia declined. These imbalances were significantly associated with higher ISS, longer ICU stays, increased ventilation needs, and prolonged hospitalization. Figure 3 presents a box plot showing the relationship between KL at ICU discharge and hospital LOS, demonstrating that patients with more severe dyskalemia required extended inpatient care. This underscores the prognostic value of potassium trends throughout the ICU course.

At **hospital discharge**, most patients showed normalized KL; however, a notable proportion still had extreme hypokalemia. Figure 4 displays a box plot comparing KLs at discharge with hospital LOS, revealing that patients with more pronounced dyskalemia experienced longer hospitalizations. Additionally, KLs at the **time of patient death** were strongly associated with a higher ISS, lower GCS, prolonged ICU and ED stays, and increased VDs, highlighting the persistent prognostic value of potassium imbalances through end-of-life care.

Table 3 reinforces the association between potassium imbalances and prolonged hospitalization. Patients with dyskalemia—particularly hypokalemia—tended to have longer hospital and ICU stays, as well as increased ventilation requirements. These trends highlight the value of potassium monitoring in anticipating care needs.

Overall, our findings underscore the prognostic importance of potassium imbalances, especially extreme hypokalemia, in severe TBI. As hospitalization progresses, these disturbances become more prevalent and are closely linked to extended ICU stays, greater ventilation use, and higher mortality. Routine potassium surveillance may support early intervention and improve outcomes in critically ill trauma patients

### 3.2. Comparison to the Existing Literature

Potassium imbalances are well-documented in trauma populations and occur in up to 50% of TBI patients [8]. Prior studies report hypokalemia in 32.4–45% of cases [4,7,9,10], which aligns with our observed rate of 12.03% on admission and progression to 39.14% in the ICU and 42.71% by discharge. These findings reflect the worsening nature of dyskalemia during critical care [8,9].

The lower rate of hypokalemia observed on admission in our cohort compared to previously reported rates (12.03% vs. 32.4–45%) may be explained by several factors. First, our exclusion criteria removed patients who died or were discharged within 24 h, potentially excluding individuals with acute or more unstable metabolic disturbances. Second, measurement timing may have played a role: potassium levels were captured based on the first lab draw in the trauma bay, which may vary slightly from timing protocols in other studies. Finally, differences in institutional triage thresholds and admission criteria could introduce selection bias by underrepresenting milder TBI cases that may have presented with early hypokalemia.

Hypokalemia is associated with serious complications such as arrhythmias, rhabdomyolysis, renal failure, and hyperglycemia [11,12]. In our cohort, patients with hypokalemia had a lower GCS and higher ISS, supporting prior evidence that links electrolyte imbalance with injury severity and poor outcomes [9,11,26].

Several studies have suggested that normalization of serum potassium may have a protective effect in trauma and critical care settings. For example, Micah Ngatuvai et al. (2023) [7] reported that prompt correction of hypokalemia in TBI patients was associated with reduced ICU length of stay and lower incidence of arrhythmias. While our dataset does not permit the evaluation of interventional effects, these findings support the hypothesis that proactive potassium management may improve outcomes—an area that warrants further prospective research.

However, our dataset did not include consistent documentation of potassium supplementation—whether administered orally or intravenously—so we were unable to assess outcome differences between patients who received potassium correction and those who did not. This represents an important limitation and highlights the need for future prospective studies to capture intervention data in order to evaluate the clinical impact of targeted potassium management in TBI care.

Severe hypokalemia and hypophosphatemia have also been identified as independent predictors of mortality in TBI [4,27], with hypophosphatemia indicating possible progression to brain death [28]. Our analysis revealed that extreme hypokalemia was significantly associated with mortality, as well as with an elevated ISS, lower GCS, longer ED/ICU stays, and greater ventilation needs.

These patterns may be driven by endogenous catecholamine surges and insulin-mediated intracellular potassium shifts, both implicated in post-injury electrolyte imbalances [29,30,31,32]. Although we did not track insulin or glucose administration, these mechanisms are relevant and warrant further investigation.

These findings raise an important clinical question: whether hypokalemia is simply a reflection of greater injury severity, or if it plays an active pathophysiological role in worsening secondary brain injury. While our data demonstrate strong correlations between hypokalemia and ISS/GCS, causality cannot be inferred from this retrospective analysis. However, mechanistic pathways, such as membrane depolarization and neurotransmitter imbalance, suggest that early hypokalemia may indeed contribute to brain injury progression—a hypothesis that warrants prospective investigation.

In summary, our study reinforces previous findings that potassium disturbances—particularly extreme hypokalemia—are prevalent, worsen over time, and predict worse outcomes in TBI patients. Continuous monitoring and timely correction of KLs may play a key role in optimizing care and reducing mortality in this population.

### 3.3. Implications of the Study Findings

This study highlights the prognostic value of potassium imbalances in severe TBI. Extreme hypokalemia was associated with prolonged ICU and hospital stays, increased ventilation duration, and higher mortality, suggesting its utility as a clinical marker for adverse outcomes.

The correlation between dyskalemia and severity indices (AIS, ISS, GCS) suggests it may reflect underlying physiological disturbances, such as catecholamine excess, metabolic shifts, and systemic inflammation [33]. Regular potassium monitoring at admission and during ICU care may help anticipate complications and guide treatment.

Importantly, nearly 30% of patients had persistent potassium imbalances at ICU discharge. This may result from complex TBI physiology, treatment effects, or shifting priorities during recovery. Continued monitoring in step-down and neurosurgical units is warranted to prevent complications.

Maintaining potassium balance could reduce risks such as arrhythmias, cardiovascular instability, and multi-organ dysfunction—all contributors to mortality [34]. The link between dyskalemia and prolonged ventilation also suggests that correction may support faster weaning and shorter ICU stays.

These findings support integrating routine potassium surveillance into TBI care protocols. The early identification and correction of dyskalemia could improve recovery and reduce critical care burden. Further research is needed to explore causal mechanisms and evaluate targeted interventions.

### 3.4. Strengths, Limitations, and Future Perspectives

This study provides novel insights into the relationship between potassium imbalances and clinical outcomes in patients with severe traumatic brain injury (TBI). By analyzing a large cohort at a level 1 trauma center, we identified significant associations between potassium levels and mortality, hospital and ICU length of stay, and mechanical ventilation duration. These findings underscore the prognostic value of potassium fluctuations and support the need for incorporating electrolyte surveillance into TBI care protocols.

Nonetheless, several limitations warrant consideration. The single-center retrospective design may limit generalizability to other institutions with different clinical practices or patient populations. Selection bias is possible due to the exclusion of patients who died or were discharged within 24 h, potentially omitting those with acute or severe metabolic instability. Although potassium levels were consistently measured at key stages of care, some values were missing at specific time points, particularly at ICU or hospital discharge. However, early potassium measurements are likely the most clinically informative, as they reflect patient status prior to major interventions.

We also lacked data on whether patients received potassium supplementation, insulin or glucose infusions, or other electrolyte-related therapies, which could influence serum potassium levels and outcomes. Similarly, coexisting imbalances in magnesium and phosphate were not assessed, although these are common in TBI and may confound the associations observed [8]. While GCS was used as a proxy for injury severity, it may not capture the full spectrum of intracranial pathology. Imaging-based scores, such as the Rotterdam CT score, could enhance future risk stratification and are recommended in subsequent studies.

Importantly, while we identified strong statistical associations between hypokalemia and adverse outcomes, our design does not allow for causal inferences. Future prospective, multicenter studies are needed to validate these findings and determine whether interventions targeting potassium normalization can improve survival, shorten ICU stay, or accelerate neurological recovery. Interventional research should also explore the impact of insulin, fluid therapy, and pharmacologic agents on potassium homeostasis.

Additionally, it would be beneficial to explore the relationship between electrolyte imbalances and specific brain injury patterns. By identifying how potassium levels correlate with particular injury types or severities—potentially characterized using radiologic tools such as the Rotterdam CT score—we may be able to tailor treatment strategies more effectively for different subsets of TBI patients. Furthermore, evaluating the role of pharmacologic interventions, such as diuretics, potassium supplements, or other electrolyte-modifying therapies, would help refine clinical management. Understanding how these treatments influence both electrolyte levels and patient recovery could lead to more precise and individualized care.

A more dynamic approach to potassium monitoring—such as more frequent or continuous sampling—may yield stronger correlations with clinical events and allow for timely therapeutic adjustments. Incorporating long-term neurological follow-up, functional outcomes, and biomarker data would also help assess the downstream effects of potassium correction, particularly since neurological recovery is the ultimate goal of TBI management [25].

In summary, our results reinforce the clinical importance of potassium regulation in TBI and offer a foundation for more targeted, personalized management strategies. Addressing the limitations noted here through rigorous, prospective study designs will be key to translating these findings into optimized care and improved outcomes for patients with severe brain injury.

## 4. Conclusions

Potassium imbalances are common in patients with severe traumatic brain injury and have important implications for prognosis and clinical management. This study shows that dyskalemia—particularly extreme hypokalemia—is strongly associated with prolonged ICU and hospital stays, greater need for mechanical ventilation, and increased mortality. These findings highlight the utility of serum potassium as a dynamic biomarker for risk stratification and clinical decision-making in the acute phase of TBI care.

While this retrospective analysis cannot establish causality, the biological mechanisms linking hypokalemia to adverse outcomes—such as membrane depolarization, metabolic dysfunction, and disruption of the blood–brain barrier—are well-supported in prior studies and remain pathophysiologically compelling [17]. The consistent associations observed across multiple care points reinforce the need for timely identification and correction of potassium disturbances in critical care protocols.

Future prospective and interventional studies are needed to determine whether active normalization of potassium levels can improve clinical trajectories and long-term recovery. Such efforts will be critical in advancing precision electrolyte management as a component of modern neurotrauma care and may contribute meaningfully to reducing the burden of secondary brain injury.

## Figures and Tables

**Table 1:** Baseline characteristics of patients with severe traumatic brain injury (TBI) included in the study.

	Frequency	Percent	Cumulative Frequency	Cumulative Percent
**Gender**				
*Female*	229	23.34	229	23.34
*Male*	752	76.66	981	100.00
**Race**				
*American Indian*	1	0.10	1	0.10
*Asian*	143	14.58	144	14.68
*Black*	76	7.75	220	22.43
*Native Hawaiian Or Another Pacific Islander*	4	0.41	224	22.83
*Other*	562	57.29	786	80.12
*Unknown*	18	1.83	804	81.96
*White*	177	18.04	981	100.00
**Ethnicity**				
*Hispanic Origin*	455	46.38	455	46.38
*Non-Hispanic Origin*	485	49.44	940	95.82
*Unknown*	41	4.18	981	100.00
**Age**				
*0 < age < 15*	17	1.76	17	1.76
*15 ≤ age < 24*	60	6.21	77	7.97
*24 <= age <= 64*	579	59.94	656	67.91
*age > 65*	310	32.09	966	100.00
**Trauma Type**				
*Blunt*	961	97.96	961	97.96
*Penetrating*	20	2.04	981	100.00
**Mortality**				
*Non-survival*	877	89.40	877	89.40
*Survival*	104	10.60	981	100.00

**Table 2:** Frequency and percentages of various KLs at different time points during hospitalization.

K Levels at Hospital Admission (ta)	Frequency	Percent	Cumulative Frequency	Cumulative Percent
**Extreme Hypokalemia—2.5 or less mEq/L**	42	4.28	42	4.28
**Hypokalemia—2.6–3.4 mEq/L**	118	12.03	160	16.31
**Normokalemia—3.5 to 5.2 mEq/L**	782	79.71	942	96.02
**Hyperkalemia—greater than 5.2 mEq/L to 7.0 mEq/L**	39	3.98	981	100.00
**krl1**	**Frequency**	**Percent**	**Cumulative** **Frequency**	**Cumulative** **Percent**
**Extreme Hypokalemia—2.5 or less mEq/L**	384	39.14	384	39.14
**Hypokalemia—2.6–3.4 mEq/L**	76	7.75	460	46.89
**Normokalemia—3.5 to 5.2 mEq/L**	501	51.07	961	97.96
**Hyperkalemia—greater than 5.2 mEq/L to 7.0 mEq/L**	20	2.04	981	100.00
**krl2**	**Frequency**	**Percent**	**Cumulative** **Frequency**	**Cumulative** **Percent**
**Extreme Hypokalemia—2.5 or less mEq/L**	419	42.71	419	42.71
**Hypokalemia—2.6–3.4 mEq/L**	37	3.77	456	46.48
**Normokalemia—3.5 to 5.2 mEq/L**	518	52.80	974	99.29
**Hyperkalemia—greater than 5.2 mEq/L to 7.0 mEq/L**	7	0.71	981	100.00
**Khd**	**Frequency**	**Percent**	**Cumulative** **Frequency**	**Cumulative** **Percent**
**Extreme Hypokalemia—2.5 or less mEq/L**	275	28.03	275	28.03
**Hypokalemia—2.6–3.4 mEq/L**	24	2.45	299	30.48
**Normokalemia—3.5 to 5.2 mEq/L**	673	68.60	972	99.08
**Hyperkalemia—greater than 5.2 mEq/L to 7.0 mEq/L**	9	0.92	981	100.00
**overallK**	**Frequency**	**Percent**	**Cumulative** **Frequency**	**Cumulative** **Percent**
**Extreme Hypokalemia—2.5 or less mEq/L**	481	49.03	481	49.03
**Hypokalemia—2.6–3.4 mEq/L**	380	38.74	861	87.77
**Normokalemia—3.5 to 5.2 mEq/L**	116	11.82	977	99.59
**Hyperkalemia—greater than 5.2 mEq/L to 7.0 mEq/L**	3	0.31	980	99.90

**Table 3:** Mean hospital LOS of various KLs at different time points during hospitalization.

Variable	Variable Levels	*N*	Mean	Std dev	Min	Max
**K TA**	Extreme Hypokalemia—2.5 or less mEq/L	41	5.2926829	6.0631836	1.0000000	26.0000000
	Hypokalemia—2.6–3.4 mEq/L	118	13.1610169	24.9632107	1.0000000	247.0000000
	Normokalemia—3.5 to 5.2 mEq/L	779	11.7175866	19.7060620	1.0000000	180.0000000
	Hyperkalemia—greater than 5.2 mEq/L to 7.0 mEq/L	39	12.3333333	16.9307154	1.0000000	94.0000000
	Extreme Hyperkalemia—higher than 7.0 mEq/L	0	0	0	0	0
**K RL1**	Extreme Hypokalemia—2.5 or less mEq/L	382	9.0628272	18.1018185	1.0000000	180.0000000
	Hypokalemia—2.6–3.4 mEq/L	75	14.0533333	30.8951555	1.0000000	247.0000000
	Normokalemia—3.5 to 5.2 mEq/L	500	13.2400000	19.2876756	1.0000000	177.0000000
	Hyperkalemia—greater than 5.2 mEq/L to 7.0 mEq/L	20	12.1500000	14.4523719	1.0000000	50.0000000
	Extreme Hyperkalemia—higher than 7.0 mEq/L	0	0	0	0	0
**K RL2**	Extreme Hypokalemia—2.5 or less mEq/L	417	8.7769784	17.0060975	1.0000000	180.0000000
	Hypokalemia—2.6–3.4 mEq/L	37	6.3243243	5.8833761	1.0000000	22.0000000
	Normokalemia—3.5 to 5.2 mEq/L	516	14.2131783	22.3532344	1.0000000	247.0000000
	Hyperkalemia—greater than 5.2 mEq/L to 7.0 mEq/L	7	21.5714286	17.7281052	3.0000000	50.0000000
	Extreme Hyperkalemia—higher than 7.0 mEq/L	0	0	0	0	0
**K HD**	Extreme Hypokalemia—2.5 or less mEq/L	274	10.3321168	17.0146217	1.0000000	155.0000000
	Hypokalemia—2.6–3.4 mEq/L	24	4.9166667	6.7367691	1.0000000	30.0000000
	Normokalemia—3.5 to 5.2 mEq/L	670	12.4283582	21.3929029	1.0000000	247.0000000
	Hyperkalemia—greater than 5.2 mEq/L to 7.0 mEq/L	9	11.4444444	11.8438920	1.0000000	36.0000000
	Extreme Hyperkalemia—higher than 7.0 mEq/L	0	0	0	0	0
**K PD**	Extreme Hypokalemia—2.5 or less mEq/L	899	11.5116796	19.6187671	1.0000000	247.0000000
	Hypokalemia—2.6–3.4 mEq/L	8	35.3750000	43.2498968	1.0000000	108.0000000
	Normokalemia—3.5 to 5.2 mEq/L	46	10.3260870	11.5682599	1.0000000	53.0000000
	Hyperkalemia—greater than 5.2 mEq/L to 7.0 mEq/L	10	19.0000000	45.5241084	1.0000000	148.0000000
	Extreme Hyperkalemia—higher than 7.0 mEq/L	14	5.8571429	5.0054915	1.0000000	15.0000000

## Data Availability

Data for this study was requested from the Elmhurst Trauma registry and extracted using electronic medical records after receiving approval from the Institutional Review Board at our facility (Elmhurst Hospital Center).
